# Altered Spike Immunoglobulin G Fc N-Linked Glycans Are Associated With Hyperinflammatory State in Adult Coronavirus Disease 2019 and Multisystem Inflammatory Syndrome in Children

**DOI:** 10.1093/ofid/ofae626

**Published:** 2024-10-16

**Authors:** Jacob D Sherman, Vinit Karmali, Bhoj Kumar, Trevor W Simon, Sarah Bechnak, Anusha Panjwani, Caroline R Ciric, Dongli Wang, Christopher Huerta, Brandi Johnson, Evan J Anderson, Nadine Rouphael, Matthew H Collins, Christina A Rostad, Parastoo Azadi, Erin M Scherer

**Affiliations:** Division of Infectious Diseases, Department of Medicine, Emory University School of Medicine, Atlanta, Georgia, USA; Division of Infectious Diseases, Department of Medicine, Emory University School of Medicine, Atlanta, Georgia, USA; Complex Carbohydrate Research Center, University of Georgia, Athens, Georgia, USA; Division of Infectious Diseases, Department of Medicine, Emory University School of Medicine, Atlanta, Georgia, USA; Division of Infectious Diseases, Department of Medicine, Emory University School of Medicine, Atlanta, Georgia, USA; Division of Infectious Diseases, Department of Medicine, Emory University School of Medicine, Atlanta, Georgia, USA; Division of Infectious Diseases, Department of Pediatrics, Emory University School of Medicine, Atlanta, Georgia, USA; Division of Infectious Diseases, Department of Medicine, Emory University School of Medicine, Atlanta, Georgia, USA; Division of Infectious Diseases, Department of Medicine, Emory University School of Medicine, Atlanta, Georgia, USA; Division of Infectious Diseases, Department of Medicine, Emory University School of Medicine, Atlanta, Georgia, USA; Division of Infectious Diseases, Department of Medicine, Emory University School of Medicine, Atlanta, Georgia, USA; Division of Infectious Diseases, Department of Pediatrics, Emory University School of Medicine, Atlanta, Georgia, USA; Division of Infectious Diseases, Department of Medicine, Emory University School of Medicine, Atlanta, Georgia, USA; Division of Infectious Diseases, Department of Medicine, Emory University School of Medicine, Atlanta, Georgia, USA; Division of Infectious Diseases, Department of Pediatrics, Emory University School of Medicine, Atlanta, Georgia, USA; Complex Carbohydrate Research Center, University of Georgia, Athens, Georgia, USA; Division of Infectious Diseases, Department of Medicine, Emory University School of Medicine, Atlanta, Georgia, USA

**Keywords:** antibody glycosylation, COVID-19, Fc, inflammation, MIS-C

## Abstract

**Background:**

Severe coronavirus disease 2019 (COVID-19) and multisystem inflammatory syndrome (MIS-C) are characterized by excessive inflammatory cytokines/chemokines. In adults, disease severity is associated with severe acute respiratory syndrome coronavirus 2 (SARS-CoV-2)–specific immunoglobulin G (IgG) Fc afucosylation, which induces proinflammatory cytokine secretion from innate immune cells. This study aimed to define spike IgG Fc glycosylation following SARS-CoV-2 infection in adults and children and following SARS-CoV-2 vaccination in adults and the relationships between glycan modifications and cytokines/chemokines.

**Methods:**

We analyzed longitudinal (n = 146) and cross-sectional (n = 49) serum/plasma samples from adult and pediatric COVID-19 patients, MIS-C patients, adult vaccinees, and adult and pediatric controls. We developed methods for characterizing bulk and spike IgG Fc glycosylation by capillary electrophoresis and measured levels of 10 inflammatory cytokines/chemokines by multiplexed enzyme-linked immunosorbent assay.

**Results:**

Spike IgG was more afucosylated than bulk IgG during acute adult COVID-19 and MIS-C. We observed an opposite trend following vaccination, but it was not significant. Spike IgG was more galactosylated and sialylated and less bisected than bulk IgG during adult COVID-19, with similar trends observed during pediatric COVID-19/MIS-C and following SARS-CoV-2 vaccination. Spike IgG glycosylation changed with time following adult COVID-19 or vaccination. Afucosylated spike IgG exhibited inverse and positive correlations with inflammatory markers in MIS-C and following vaccination, respectively; galactosylated and sialylated spike IgG inversely correlated with proinflammatory cytokines in adult COVID-19 and MIS-C; and bisected spike IgG positively correlated with inflammatory cytokines/chemokines in multiple groups.

**Conclusions:**

We identified previously undescribed relationships between spike IgG glycan modifications and inflammatory cytokines/chemokines that expand our understanding of IgG glycosylation changes that may impact COVID-19 and MIS-C immunopathology.

Severe acute respiratory syndrome coronavirus 2 (SARS-CoV-2) infections have a wide range of coronavirus disease 2019 (COVID-19) severities. While many adults infected with SARS-CoV-2 now experience mild COVID-19, during the early phase of the pandemic, approximately 14% and 5% of adult patients developed severe or critical COVID-19, respectively [1]. Children and young adults generally present with mild symptoms, but occasionally can have severe disease, and early in the pandemic, 1 of 3200 pediatric SARS-CoV-2 infections developed into a severe inflammatory disease termed multisystem inflammatory syndrome in children (MIS-C) [2]. MIS-C is considered life threatening [3], though fortunately its incidence seems to be decreasing over time [4, 5]. Severe adult COVID-19 and MIS-C are characterized by excessive inflammation, including elevated inflammatory cytokines and chemokines such as tumor necrosis factor alpha (TNF-α), interleukin (IL) 6, IL-10, and IL-1β [1, 6–16]. The precise cause of these hyperinflammatory states is incompletely understood [8, 9].

An area of increasing investigation is whether irregular immunoglobulin G (IgG) Fc glycosylation is triggering dysregulated inflammation in COVID-19 and MIS-C [17–24]. The IgG Fc region contains a conserved asparagine(N)-linked glycosylation site at N297 on each heavy chain [25]. This glycan is composed of a core 7 saccharides containing 3 mannose and 4 N-acetylglucosamine (GlcNAc) residues that can be differentially modified with other sugar residues [25] (Figure 1). This diversity of structures has implications for an Fc's interaction with Fc gamma receptors (FcγRs), which can have downstream signaling effects on an active immune response. For example, it has been demonstrated that afucosylated IgG Fcs have an increased affinity for FcγRIIIa, an activating FcγR found on the surface of monocytes, macrophages, and natural killer cells, as well as FcγRIIIb, an activating FcγR found on neutrophils [26–30]. The abundance of afucosylated IgG rises in multiple viral infections, including human immunodeficiency virus, cytomegalovirus, and dengue [23, 31]. Afucosylated spike IgG1 is also more prevalent in severe than mild cases of COVID-19 [17, 19, 21, 23], with elevated afucosylated IgG1 predicting disease progression [17]. Moreover, afucosylated SARS-CoV-2 spike IgG immune complexes stimulate ex vivo human monocytes to secrete inflammatory cytokines [21] and drive inflammatory responses in mouse models expressing human FcγRs [17].

**Figure 1.**
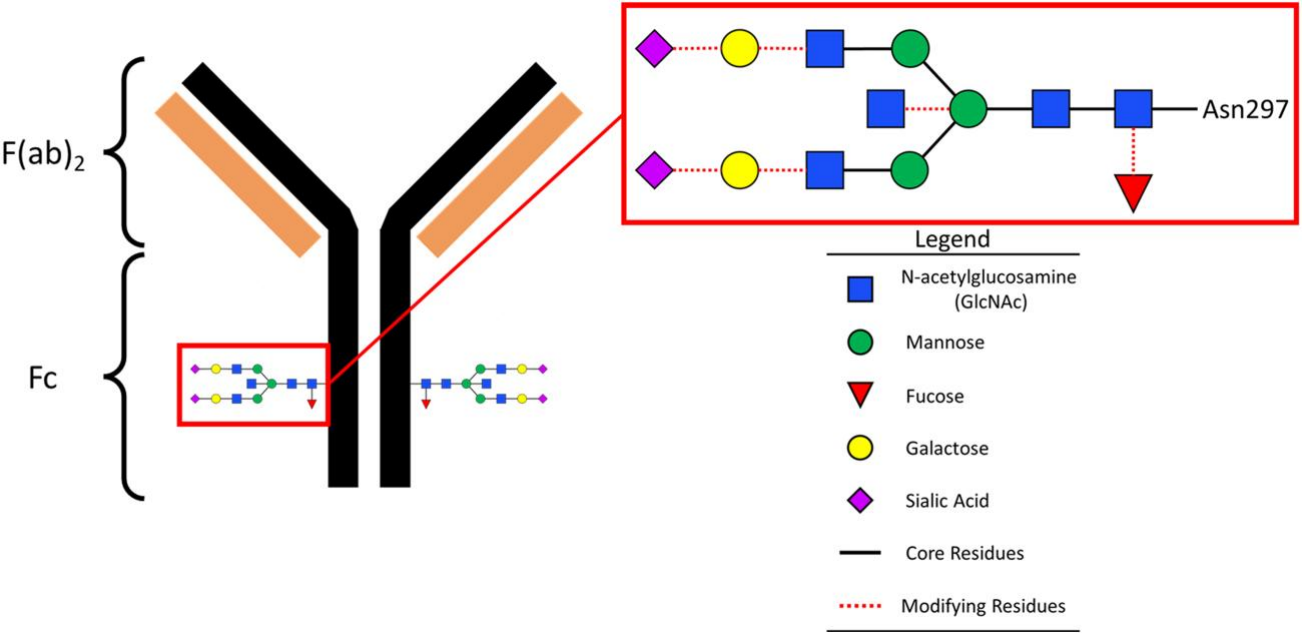
Schematic of immunoglobulin G (IgG) structure with conserved N-linked complex glycan at residue 297. IgG is composed of the antigen-binding domains, F(ab)_2_, and constant domain, Fc. The Fc has a highly conserved complex N-glycan at Asn297, which is composed of a core with 7 GlcNAc and mannose sugars and additional, variable modifying sugars.

The role of other N297 glycan modifications in inflammation, namely sialylation, galactosylation, and bisection, remains less clear [25]. Studies indicate that Fc sialylation and galactosylation are anti-inflammatory and that bisecting GlcNAc is positively associated with inflammation [25, 32–34]. However, the findings related to sialylation are discordant among studies and definitive causal relationships between these glycan modifications and changes in inflammation are lacking [25, 35]. Prior work has found SARS-CoV-2 spike IgG to be more galactosylated and sialylated and less bisected than bulk IgG from the same specimens [19, 23]. These studies have also found that bulk and spike IgG are generally less galactosylated, sialylated, and bisected in severe COVID-19 than mild COVID-19, though these comparisons did not always reach significance [19, 23].

Taken together, these studies suggest that afucosylated spike IgG plays a role in inflammation during severe COVID-19, and we hypothesized this may also extend to MIS-C because of its postinfectious hyperinflammatory profile. When this study was initiated, it was also unknown whether SARS-CoV-2 vaccination led to similar changes in spike IgG glycosylation. The primary objective of this study was to determine whether glycosylation changes are simply byproducts of the antigen-specific response and thus also stimulated by vaccination, or if glycosylation changes are unique to MIS-C and COVID-19 and thus potentially driving severe inflammation.

## MATERIALS AND METHODS

A comprehensive description of materials and methods for this study is included in the Supplementary Information.

### Patient Consent

Patients' written consent was obtained for collection of specimens for secondary use. Use of the described secondary research samples for this study was approved by the Emory University Institutional Review Board (STUDY00002583). A breakdown of the study groups is shown in Supplementary Table 1.

### Cytokine/Chemokine Multiplex Enzyme-Linked Immunosorbent Assay

Cytokine and chemokine levels were quantified using a custom Meso Scale Discovery U-plex assay.

### Bulk IgG Isolation, IdeZ Digest, and Fc Enrichment

Heat-inactivated serum or plasma samples (200 µL [36]) were desalted with Zeba Spin Desalting plates (ThermoFisher) and bulk IgG purified using Melon Gel Spin plates (ThermoFisher). Twenty-two microliters of purified bulk IgG was separated into F(ab)_2_ and Fc fragments by digestion with IdeZ protease (New England Biolabs). Bulk IgG digests were incubated with Protein G Dynabeads to enrich IgG Fc, and Fc was eluted by adding 0.1 M citric acid (pH 3.0) neutralized with 1 M sodium carbonate-bicarbonate buffer (pH 10.8).

### Anti-SARS-CoV-2 Spike IgG Isolation and IdeZ Digest

Anti-SARS-CoV-2 spike IgG enrichment was performed with 50 µL His-tag Dynabeads (Invitrogen 10104D) coated with 12 µg His-tagged recombinant SARS-CoV-2 spike in 3 rounds. Remaining bulk IgG samples were applied to Dynabead-spike complexes and supernatants transferred to the next set of beads (for second and third enrichments) or discarded (after the third enrichment). Beads from each round of enrichment were pooled for Fc digestion with IdeZ. These IdeZ digests proceeded directly to PNGaseF digest.

### PNGaseF Digest

N-linked deglycosylation of bulk or spike IgG Fc was performed with Rapid PNGaseF (New England Biolabs). After incubation, PNGaseF digests were allowed to dry completely overnight in a biosafety cabinet. Once dried, plates were sealed and stored at 4°C until fluorescent labeling.

### Fluorescent Labeling of IgG Fc Glycans

Dried PNGaseF digests were reconstituted in molecular biology–grade water. A solution of 20 mM 8-aminopyrene trisodium salt (APTS) (Sigma-Aldrich) and 3.6 M citric acid (Sigma-Aldrich) was prepared and mixed in equal volume with 200 mM 2-picoline borane (Sigma-Aldrich) in dimethyl sulfoxide (Sigma-Aldrich). This solution was added to each sample and incubated at 37°C for 16 hours, then quenched with 20% ultra-purified water, 80% acetonitrile (Sigma-Aldrich) (v/v). Hydrophilic interaction chromatography–based solid phase extraction (HILIC-SPE) was performed to isolate APTS-labeled N-glycans from other reagents as described previously [37]. APTS-labeled samples were stored at −20°C until capillary electrophoresis (CE) analysis.

### Capillary Electrophoresis

APTS-labeled samples were analyzed using an ABI3130XL or ABI3500XL instrument (Applied Biosystems) with an APTS-labeled N-glycan reference panel (Agilent) run in parallel.

Resulting electropherograms were analyzed using GeneMarker (v3.0.1). Area under the curve (AUC) was quantified for each peak to determine glycoform abundance out of total glycan AUC.

### Mass Spectrometry

An IgG Fc sample (5 µg) was desalted and further reduced and alkylated with 5 mM dithiothreitol and 10 mM iodoacetamide. Proteins were digested with a 1:20 ratio of trypsin at 37°C for 18 hours. The resulting peptides were subjected to liquid chromatography with tandem mass spectrometry (LC-MS/MS) on an Orbitrap Eclipse mass spectrometer (ThermoFisher) equipped with 3000 RSLCnano system. Data were acquired in positive ion mode and processed with Byonic software (Protein Metrics; v4.0.12) and searched against the IgG Fc sequence and a catalogue of >59 human N-linked glycans. The relative percentages of each glycoform were determined by deconvolution of the LC-MS data at the full MS level, then determining the AUC for each full MH+.

### Statistical Analysis

Statistical analyses were performed in GraphPad Prism (version 9.4.1).

## RESULTS

We evaluated cytokine/chemokine levels, bulk IgG Fc glycosylation, and spike IgG Fc glycosylation in sera/plasma specimens from cross-sectional or longitudinal groups of adult healthy controls (n = 10), COVID-19 patients (n = 109), and messenger RNA (mRNA) SARS-CoV-2 vaccine study participants (n = 37), as well as cross-sectional groups of pediatric healthy controls (n = 10) and COVID-19 (n = 9) and MIS-C patients (n = 20) (Supplementary Table 1). The adult COVID-19 group was comprised of patients with severity ranging from moderate (ordinal scale [OS] 4) to severe and critical (OS5 and OS7). COVID-19 patient samples were collected during acute hospitalization for COVID-19 (at enrollment; ≤12 days post–symptom onset) and during convalescence (29 days post enrollment), when infection-associated spike IgG is at its peak [38]. Acute adult COVID-19 samples, acute pediatric COVID-19 samples, and MIS-C samples were first analyzed by enzyme-linked immunosorbent assay (ELISA) for seropositivity to SARS-CoV-2 spike protein, with 89 of 109 (82%), 8 of 9 (88.9%), and 20 of 20 (100%) considered seropositive, respectively, by criteria described in the extended Materials and Methods section of the Supplementary Information. These results are consistent with the timing of specimen collection (Supplementary Table 1) and published observations that spike IgG levels develop in the first 2 weeks of symptom onset for both adults and children [22, 38], whereas MIS-C patients typically exhibit high spike IgG levels upon MIS-C symptom onset [9, 14]. Thirty-two percent (12/37) of adult vaccinee samples were seropositive at baseline (7/19 [36%] who received mRNA-1273 and 5/18 [27.8%] who received BNT162b2). Seronegative acute samples were omitted from spike IgG Fc glycosylation analysis.

### Cytokine Analysis

**Figure 2.**
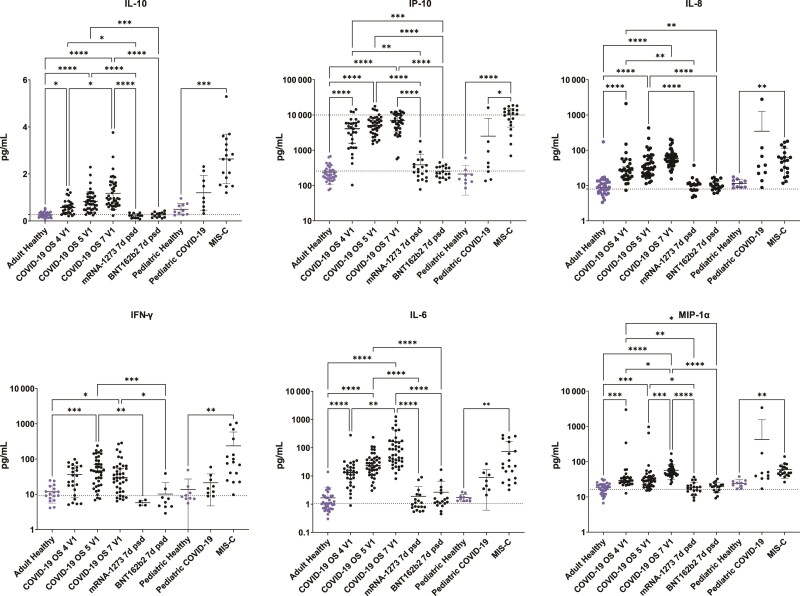
Proinflammatory cytokines and chemokines are significantly elevated in adult coronavirus disease 2019 (COVID-19) patients and multisystem inflammatory syndrome (MIS-C) patients compared to healthy controls. Differences in interleukin (IL) 10, interferon gamma induced protein 10 (IP-10), IL-8, interferon gamma (IFN-γ), IL-6, and macrophage-inflammatory protein 1 alpha (MIP-1α) levels between adult healthy controls, which includes vaccinees at baseline, patients enrolled in the Adaptive COVID-19 Treatment Trial (ACTT-1) with different COVID-19 severities by ordinal scale (OS) at enrollment (V1), or vaccinees (7 d post–second dose [7d psd]); as well as between pediatric healthy controls or pediatric patients with severe acute respiratory syndrome coronavirus 2 or MIS-C. Kruskal-Wallis with Dunn test (*P < .05, **P < .01, ***P < .001, ****P < .0001).

We evaluated systemic inflammatory cytokine and chemokine (IL-10, interferon gamma induced protein 10 [IP-10]/CXCL10, IL-8/CXCL8, interferon gamma [IFN-γ], IL-6, macrophage-inflammatory protein 1 alpha [MIP-1α]/CCL3, IL-1β, monocyte chemoattractant protein 1 [MCP-1]/CCL2, monocyte chemoattractant protein 3 [MCP-3]/CCL7, and TNF-α) levels in all specimens. Adult acute COVID-19 patients exhibited significantly increased cytokine/chemokine levels compared to healthy controls and SARS-CoV-2 mRNA vaccinees for 9 of 10 of the markers measured, but not IL-1β (Figure 2, Supplementary Figure 1). IL-10, IL-6, MIP-1α, MCP-1, MCP-3, and TNF-α were significantly elevated in critical COVID-19 (OS7) compared to moderate COVID-19 (Figure 2, Supplementary Figure 1). During convalescence, cytokine/chemokine levels generally approached healthy control baseline levels, and this decrease was significant in most cases for adults with severe COVID-19 who received either remdesivir or placebo, as well as in adults with moderate COVID-19 who received remdesivir (Supplementary Figures 2 and 3). Among vaccine groups, serum cytokine levels did not significantly change between baseline and 7 days post–second vaccine dose, except for a significant increase in IP-10 (Supplementary Figure 4).

Among pediatric study groups, MIS-C patients exhibited significantly increased IL-10, IP-10, IL-8, IFN-γ, and MIP-1α compared to healthy controls (Figure 2).

### IgG Fc Glycan Analysis

**Figure 3.**
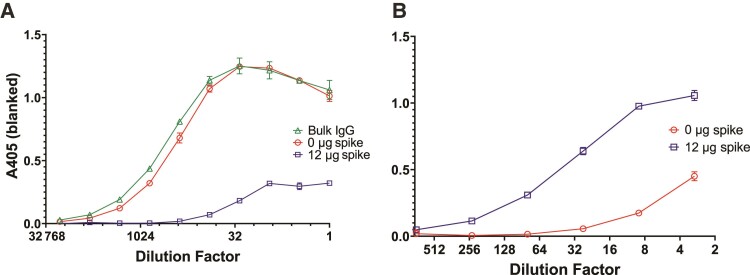
Spike-coated beads capture anti-spike immunoglobulin G (IgG) from purified bulk IgG of a seropositive participant. *A*, After treatment 3 times with His Dynabeads coated with 0 μg (red circles) or 12 μg (blue squares) severe acute respiratory syndrome coronavirus 2 spike protein, depleted bulk IgG was analyzed for spike binding by enzyme-linked immunosorbent assay alongside the purified bulk IgG (green triangles). *B*, Measurement of spike binding by postenrichment IgG eluates from His Dynabeads coated with 0 μg (red circles) or 12 μg (blue squares) spike.

To evaluate whether IgG Fc glycosylation was altered following SARS-CoV-2 infection or mRNA vaccination, we developed methods for analyzing IgG Fc glycosylation by adapting previously published methods [37, 39, 40]. We first purified bulk IgG from serum and then digested it with IdeZ protease to separate the F(ab)_2_ region from the Fc region. We did this to reduce confounding results from glycosylation in the IgG F(ab)_2_ region, which occurs in approximately 30% of F(ab)_2_ domains [25]. Protein G beads enriched the proportion of Fc domain in the sample but did not remove F(ab)_2_ domain completely (Supplementary Figure 5). To purify spike IgG for antigen-specific IgG Fc glycan analysis, we treated bulk IgG 3 times with spike-coated beads. We confirmed that spike IgG was depleted from bulk IgG by ELISA (Figure 3*A*) and that eluted spike IgG bound to spike (Figure 3*B*). To isolate the Fc domain from spike IgG, we treated IgG bound to spike-coated beads with IdeZ protease, releasing pure Fc and IdeZ with minimal contaminating F(ab)_2_ as confirmed by sodium dodecyl sulphate–polyacrylamide gel electrophoresis (Supplementary Figure 6). We then compared the concordance of bulk IgG Fc glycosylation results generated by our CE method with those generated by LC-MS/MS using biological replicate samples, where LC-MS/MS selectively identifies glycans at position N297 (ie, does not include N-linked glycans in the F(ab)_2_ domain). To compare our CE data that did not differentiate by IgG subclass to LC-MS/MS data that identified glycans abundances per IgG1, IgG2, or IgG3/4, we generated a weighted average for each glycan abundance across all subclasses by normalizing against the subclass abundance. This comparison showed good general agreement between results obtained by both methods, indicating minimal differences despite F(ab)_2_ contamination in our bulk IgG Fc preparation, though we found a higher proportion of galactosylated glycans by CE (Table 1). Potential contributors to this discrepancy in glycan proportions include the small amount of contaminating F(ab)_2_ contributing glycans to the sample, bias toward particular glycan structures during HILIC-SPE purification [41], or ionization efficiency of different glycans during LC-MS/MS [42]. Independent analyses of replicate bulk IgG Fc samples by CE showed general reproducibility, with a 2%–3% standard deviation, though low abundance glycans did not reach the cutoff for quantification when overall signal was low, which can lead to potential underestimation of low-abundance glycans as demonstrated in our data (Supplementary Figure 7).

**Table 1.** Capillary Electrophoresis Recapitulates Sialylated and Bisected Bulk Immunoglobulin G Fc Glycan Abundances Found by Liquid Chromatography With Tandem Mass Spectrometry in Biological Replicate Analysis

	Weighted Average of MS Glycan Abundance for n = 1 Biological Replicate	Median CE Glycan Abundance for n = 2 Biological Replicates (Range)
Fucosylated	99.9%	97.4% (95.3%–99.6%)
Bisected	6.7%	4.8% (2.7%–6.9%)
Galactosylated	45.4%	61.3% (55.8%–66.8%)
Sialylated	8.2%	10.1% (4.6%–15.6%)

Abbreviations: CE, capillary electrophoresis; MS, mass spectrometry.

We then analyzed bulk IgG Fc glycans by CE for all samples and spike IgG Fc glycans for seropositive acute and baseline samples, as well as all convalescent and postboost samples. We grouped glycans into 4 standardized categories: absence of core fucose (afucosylated), presence of terminal galactose (galactosylated), presence of terminal sialic acid (sialylated), or presence of bisecting GlcNAc (bisected). Four glycoforms did not separate adequately by CE, and we observed peaks in samples that were not present in our glycan standards. Thus, we conservatively determined ranges of glycan abundances for each sample, with the minimum limited to only positively identified peaks and maximum including ambiguous peaks. The median of this range was used for statistical comparisons.

**Figure 4.**
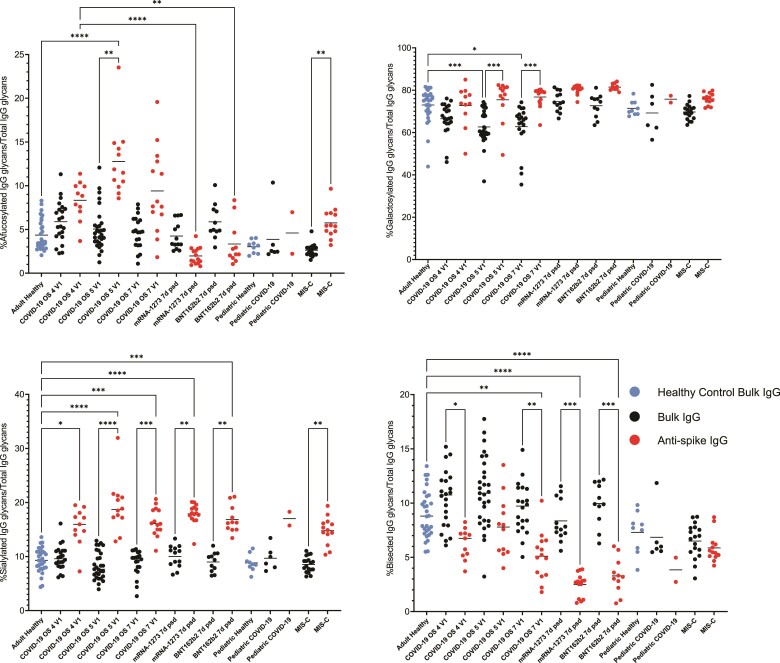
Afucosylated anti-spike immunoglobulin G (IgG) is significantly increased in severe coronavirus disease 2019 (COVID-19) and multisystem inflammatory syndrome (MIS-C) over bulk and healthy controls, while anti-spike IgG is globally more sialylated and less bisected than bulk or healthy control IgG. Kruskal-Wallis with Dunn test (*P < .05, **P < .01, ***P < .001, ****P < .0001).

Overall, we found that spike IgG was more afucosylated in acute COVID-19 than bulk IgG from the same groups, than bulk IgG of healthy controls, or than spike IgG at 6–15 days postboost, which is during the peak immune response to SARS-CoV-2 mRNA vaccination [43]. Specifically, we observed that spike IgG Fc N-linked glycans were significantly more afucosylated in severe OS5 COVID-19 compared to bulk IgG Fc glycans from the same group or adult healthy controls (Figure 4). The proportion of afucosylated spike IgG in moderate COVID-19 was also greater than that observed in BNT162b2 or mRNA-1273 vaccinees. We observed that spike IgG was more afucosylated in moderate or critical (OS7) COVID-19 than bulk IgG from the same groups or adult healthy controls, but these differences were not significant. Spike IgG in MIS-C patients was also more afucosylated than bulk IgG from the same group or bulk IgG of pediatric healthy controls, but the latter did not reach significance (Figure 4).

In contrast to the opposing trend in spike IgG Fc afucosylation observed during COVID-19/MIS-C compared to vaccination, we found that spike IgG in adult COVID-19 patients and vaccinees shared the same trends in sialylation, galactosylation, and bisection. Namely, spike IgG in adult COVID-19 patients and vaccinees was generally more sialylated and galactosylated and less bisected than bulk IgG from the same groups or healthy controls. Similarly, among pediatric groups, we observed that SARS-CoV-2 and MIS-C patients' spike IgG glycans were on average more sialylated and galactosylated and less bisected than bulk IgG glycans from the same groups or healthy controls, though these differences did not always reach significance (Figure 4).

**Figure 5.**
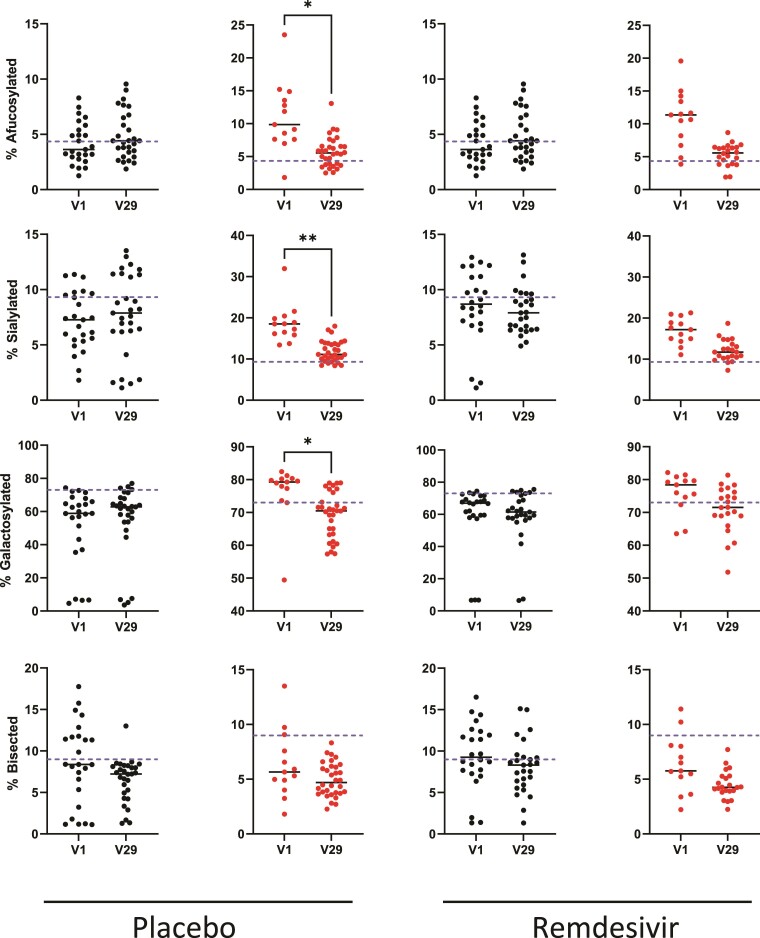
Changes in immunoglobulin G (IgG) Fc glycans between acute infection and convalescence in patients with severe coronavirus disease 2019 (COVID-19). Differences in afucosylated, sialylated, galactosylated, or bisected bulk (black symbols, first and third columns) or anti-spike (red symbols, second and fourth columns) IgG Fc glycan abundances between acute infection and convalescence in paired serum samples of adults with severe COVID-19 (ordinal scale 5 and 7), who received either remdesivir or placebo. Wilcoxon matched-pairs signed-rank test (*P < .05, **P < .01).

Between acute COVID-19 and convalescence, the proportion of afucosylated, sialylated, and galactosylated spike IgG significantly decreased in severe COVID-19 patients who received placebo (Figure 5). Similar decreases were also observed for bisected spike IgG and among patients with severe COVID-19 who received remdesivir, but these changes did not reach significance. In patients with moderate COVID-19, the same trends were observed but were not significant (Supplementary Figure 8), possibly due to the smaller sample number in the moderate groups. The exception was that in moderate COVID-19 patients treated with remdesivir, galactosylated spike IgG abundance trended toward a slight increase between acute disease and convalescence. One possible explanation for this difference is that spike IgG was not as galactosylated in this group during the acute phase as spike IgG in severe COVID-19 patients or moderate COVID-19 patients receiving placebo (Supplementary Figure 8).

In general, there was less change in bulk IgG Fc glycosylation between acute COVID-19 and convalescence in all adult COVID-19 groups (Figure 5, Supplementary Figure 8). There were no differences in overall glycan abundance levels between adult male and female groups during acute SARS-CoV-2 infection, apart from higher bulk IgG Fc galactosylation in male patients compared to female patients (Supplementary Figure 9). There were also no significant correlations between oropharyngeal viral load as determined by reverse-transcription polymerase chain reaction [44] and bulk or spike IgG Fc glycosylation (Supplementary Figure 10). Sialylation (in bulk IgG) and galactosylation levels (in both bulk and spike IgG) significantly decreased with age, whereas both bulk and spike IgG Fc bisection levels increased with age (Supplementary Figure 10). Age-associated changes in IgG Fc glycosylation, including decreasing galactosylation and increasing bisection, are known to occur [45].

We observed different trends in spike IgG Fc glycosylation between baseline and 7 days post–second dose in vaccinees, namely, no change in sialylation, significantly increased galactosylation, and significantly decreased bisection following vaccination (Supplementary Figure 11). At the same time, we found a similar significant decrease in spike IgG Fc afucosylation between baseline and 7 days post–second dose in vaccinees as observed between acute and convalescent COVID-19, though the baseline abundance of afucosylated spike IgG in vaccinees was lower than that in acute COVID-19.

### Correlation Analysis

**Figure 6.**
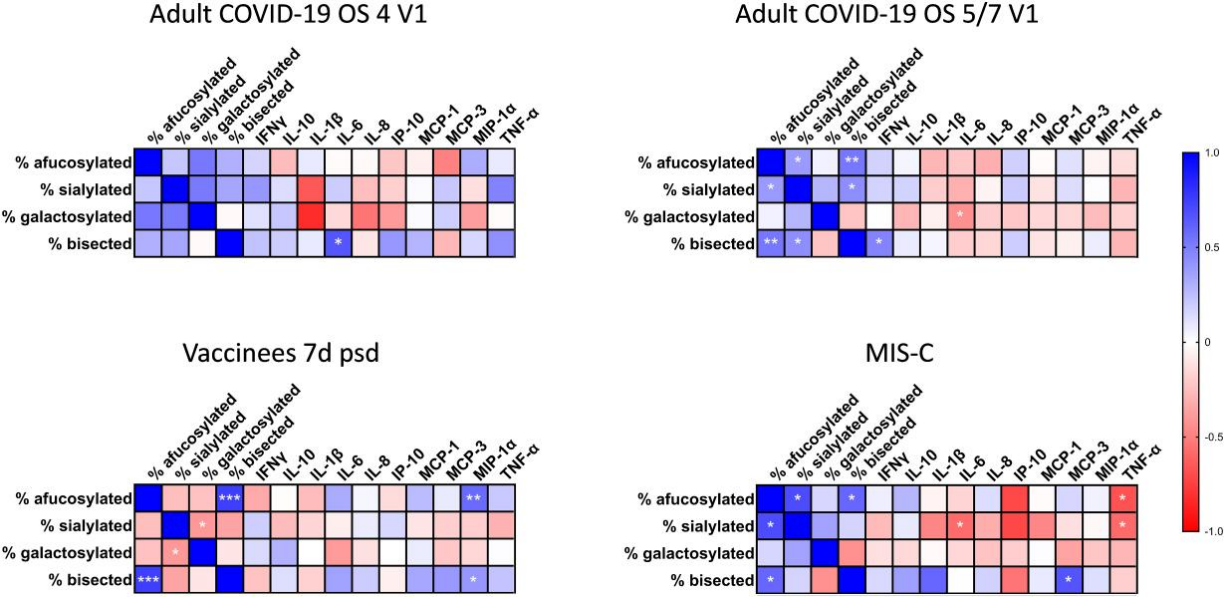
Inverse correlations observed between afucosylated, sialylated, or galactosylated glycan abundances and proinflammatory cytokine levels in adult coronavirus disease 2019 (COVID-19) and multisystem inflammatory syndrome (MIS-C) patients are not observed in vaccinees. Spearman correlation coefficients were estimated between serum cytokine/chemokine levels and spike immunoglobulin G (IgG) glycan abundances in moderate (ordinal scale [OS] 4) and severe (OS5/7) COVID-19 at enrollment (V1), mRNA vaccine recipients at 7 d after the second vaccine dose (7d psd), and MIS-C patients. Gradient from blue to red indicates strength of positive or inverse correlation, respectively. Significant relationships are indicated by asterisks (*P < .05, **P < .01, ***P < .001). Cytokine abbreviations: IFN-γ, interferon gamma; IL, interleukin; IP-10, interferon gamma induced protein 10; MCP-1, monocyte chemoattractant protein 1; MCP-3, monocyte chemoattractant protein 3; MIP-1α, macrophage-inflammatory protein 1 alpha; TNF-α, tumor necrosis factor alpha.

We then evaluated relationships between spike IgG glycan abundances and inflammatory cytokine/chemokine levels (Figure 6). We observed a positive correlation between bisected spike IgG abundances and IL-6 levels during acute moderate COVID-19 in adults. During acute severe COVID-19 in adults, we observed a negative correlation between galactosylated spike IgG abundances and IL-6 levels and a positive correlation between bisected spike IgG abundances and IFN-γ levels. In vaccinees, both afucosylated and bisected spike IgG abundances correlated positively with MIP-1α levels. For MIS-C patients, both afucosylated and sialylated spike IgG abundances correlated inversely with TNF-α levels. Sialylated spike IgG also correlated inversely with IL-6 levels in MIS-C patients. Finally, bisected spike IgG abundances in MIS-C patients positively correlated with MCP-3 levels.

## DISCUSSION

This study contributes to a growing body of work aiming to understand how IgG Fc glycosylation impacts antibody effector function, particularly as it relates to COVID-19 immunopathology. All adult COVID-19 patient groups had increased levels of inflammatory cytokines and chemokines in sera during acute COVID-19 compared to healthy controls, as other studies have found [8, 46]. Also similar to other published data [17–19, 21, 23], we found increased proportions of afucosylated spike IgG Fc glycans in COVID-19 patients during acute infection, but not following SARS-CoV-2 mRNA vaccination. While we found significant differences in the abundances of spike IgG Fc glycans with other modifications (ie, sialylation, galactosylation, bisection), only changes in spike IgG Fc afucosylation seemed to be unique to SARS-CoV-2 infection. For this reason, and as a result of prior in vitro and in vivo data linking IgG afucosylation with increased affinity for FcγRIIIa and FcγRIIIb [26, 28, 29], increased proinflammatory cytokine secretion [17, 21], increased monocyte and neutrophil recruitment [17], and increased expression of FcγRIIIa on monocytes of patients that progressed to severe COVID-19 [17], spike IgG afucosylation is of particular interest for understanding COVID-19 immunopathology. At the same time, we found that both inflammatory cytokine/chemokine levels and spike glycan abundances generally returned to healthy control values at convalescence, signaling a short half-life for these infection-associated changes. Notably, while we and others find that the abundance of afucosylated spike IgG declines from acute infection into convalescence and from baseline to post–second vaccine dose [18, 19, 23], not all groups observe this trend [17, 21, 47, 48], which may reflect differences in cohorts or timing of specimen collection. While inflammatory markers broadly correlated with altered spike IgG Fc glycan abundances, our correlation analyses identified only a few significant relationships between cytokine levels and spike glycan abundances in adult acute COVID-19 groups: a negative correlation between galactosylation and IL-6 (OS5/7), and positive correlations between bisection and IL-6 (OS4) and IFN-γ (OS5/7). Siekman et al also found significant negative correlations between spike IgG1 Fc galactosylation and IL-1β, IL-6, and IL-8 levels and spike IgG1 Fc sialylation and IL-6 levels [19]. These observations align with the theory that galactosylation is generally anti-inflammatory and bisection is generally proinflammatory [25, 32] and suggest that glycosylation modifications may be differentially impacting inflammatory cytokine expression and thus inflammation in COVID-19. Conversely, there is evidence that cytokines (eg, IFN-γ, IL-6, or IL-21) can directly modify B-cell glycosyltransferase expression and IgG Fc glycosylation, though the outcome of a particular cytokine exposure appears to depend on the system of inquiry [49–51]. This is likely because the mechanisms by which the B-cell environment impacts IgG glycosylation are still incompletely understood. Following vaccination, we found that afucosylated and bisected spike IgG abundances correlated with MIP-1α levels, all of which were very low.

Pediatric MIS-C patients also had elevated inflammatory cytokine and chemokine levels in our study. Like adult COVID-19 patients, MIS-C patients had increased proportions of afucosylated spike IgG Fc glycans, which has not been previously published. Similar to adult COVID-19 results, we identified significant correlations between glycosylation patterns and inflammatory markers in paired MIS-C samples, including a negative correlation between afucosylated spike IgG Fc glycans and TNF-α, negative correlations between sialylated glycans and TNF-α and IL-6, and a positive correlation between bisected glycans and MCP-3. While this is consistent with observed anti-inflammatory activity of IgG Fc sialylation and the proposed inflammatory activity of bisection [25, 33, 34, 52], the negative correlation between spike IgG afucosylation and TNF-α does not align with the theory that afucosylation is generally inflammatory. Still, Siekman et al also found a negative relationship between afucosylation and TNF-α in adult COVID-19 patients, though in this case it was not statistically significant [19]. Whether these relationships are biologically relevant will require follow-up studies. Longitudinal samples prior to the onset of MIS-C as well as after resolution would enable us to determine whether the observed glycosylation patterns precede onset of the condition or return to healthy control levels upon recovery, as has been established with severe adult COVID-19 [17, 19, 23]. Previous work investigating altered IgG glycosylation in MIS-C patients is limited, with our literature searches only finding a paper from Bartsch et al, who found both decreased galactosylation and fucosylation of bulk IgG Fc in severe MIS-C samples compared to pediatric healthy controls [22]. Notably, Bartsch et al did not examine antigen-specific IgG Fc glycosylation in the context of MIS-C as we did here [22]. As part of the same work, Bartsch et al note that severe MIS-C patients had increased monocyte activation [22]. Increased stimulation of FcγRIII by afucosylated antigen-specific IgG may thus be a contributing factor in the systemic inflammatory state characteristic of MIS-C; thus, our observations warrant more detailed investigation.

Much of the published research on IgG glycosylation in COVID-19 utilizes mass spectrometry for identifying glycan structure patterns in samples [18–20, 22, 24]. However, this method has some limitations, including sensitivity, throughput, and cost. Overall, analyzing antigen-specific IgG Fc glycosylation by CE worked well for our study, as the method is high-throughput, more cost effective, and highly consistent between separate runs of the same sample in our hands. While results between biological replicates analyzed by both CE and mass spectrometry were also generally consistent, the proportions of galactosylated Fc glycans were found to be higher by CE than by LC-MS/MS. Likely contributing to this discordance was the fact that not all glycans in our panel could be adequately separated by CE [45, 53], and positive glycan identification was limited to the contents of our panel of 17 glycans, though more numerous than the total number of glycoforms reported by other groups using mass spectrometry (n = 14) [18]. We were able to generate usable glycan data for 159 of 282 (56%) spike IgG and 232 of 341 (68%) bulk IgG samples, with the signal being too low for the rest, meaning samples showed identifiable glycan peaks but the peaks' AUC were below the linear range of detection. We confirmed that bulk IgG was successfully purified from all specimens, as indicated by absorbance readings (data not shown); thus, losses may have occurred during subsequent glycan digestion and labeling steps. Regarding spike IgG with no glycan results, additional purification steps were required to obtain spike IgG, and lower yields were anticipated, given the lower concentration of spike IgG in blood relative to bulk IgG. These considerations may explain the lower proportion of spike IgG glycan data that met the threshold for analysis. Additionally, because CE quantifies glycans and is thus agnostic of IgG subclass, CE is not suitable for IgG subclass–specific Fc glycosylation analysis. In contrast, LC-MS/MS enables subclass-specific Fc glycosylation analysis but suffers from lower sensitivity than CE [53]. Glycan engineering allows for precise control of the glycoform present at N297 for any IgG subclass and is our choice for subsequent mechanistic testing [54]. Still, groups utilizing mass spectrometry to analyze Fc glycosylation changes following SARS-CoV-2 infection and mRNA vaccination found similar trends across different IgG subclasses, with the exception of afucosylation of IgG3/4 after vaccination [17, 18]. Because of these limitations of CE, it is possible there were differences in spike IgG Fc glycosylation between groups or correlations between glycans and cytokines that were not captured by our data, though our results generally agree with those from similar studies [17–19, 21, 23]. Such inherent limitations of CE for analyzing antibody glycan structures emphasize its utility for observing broad trends in IgG glycosylation patterns using large numbers of samples. An additional limitation of our study is that we only looked at vaccine recipient samples after the second dose of the vaccine, so we may have missed glycosylation changes or cytokine responses present after the priming dose. For example, though other groups generally observed similar spike IgG Fc glycosylation trends following the first and second SARS-CoV-2 mRNA vaccine doses [18, 47, 48], Van Coillie et al found a transient dip in spike IgG1 fucosylation in COVID-19–naive mRNA vaccinees after the priming dose that other groups did not observe, potentially due to earlier and more frequent specimen collection [18, 47, 48].

Our findings on the alteration of antigen-specific IgG glycosylation patterns following SARS-CoV-2 infection or vaccination corroborate existing studies. Regarding MIS-C, however, our results point toward previously underexplored relationships that may be impacting the disease. Our observations warrant further investigation, which could include considerations of IgG titer, subclass, and temporal analysis of spike IgG Fc glycosylation over MIS-C and COVID-19 disease course, including in long COVID. Additional in vitro and in vivo work using glycan-engineered monoclonal antibodies with specific glycan modifications to stimulate monocytes or neutrophils for assessment of inflammatory cytokine/chemokine secretion might further elucidate the contributions of afucosylation, galactosylation, bisection, and sialylation to these effector functions. As patterns of antigen-specific IgG Fc afucosylation seem to be consistent across diverse viral infections, such studies could have broad applicability for understanding drivers of inflammation during viral infections [23, 31].

## Supplementary Material

ofae626_Supplementary_Data

## Supplementary Data

Supplementary materials are available at *Open Forum Infectious Diseases* online. Consisting of data provided by the authors to benefit the reader, the posted materials are not copyedited and are the sole responsibility of the authors, so questions or comments should be addressed to the corresponding author.

## Notes

***Acknowledgments.*** We thank Ryan McCool and Cory Acreman from the McLellan Lab (University of Texas, Austin) for providing the SARS-CoV-2 HexaPro spike [55]. We also thank Natalia Kozak-Muiznieks, Melisa Willby, and Lesley McGee from the Centers for Disease Control and Prevention (CDC) for generously allowing our use of their ABI3130XL and ABI3500XL and for their support with using the equipment; Teresa Snyder-Leiby from SoftGenetics LLC for her guidance in using the GeneMarker program for our glycan data analysis; Yongxian Xu for cytokine data analysis; Daniel Espinoza for sample processing and management; and Camila Ornelas and Michael Zianni from ThermoFisher Scientific for CE troubleshooting and protocol support.

***Author contributions*.** Study conception: E. M. S. Funding acquisition: P. A. and E. M. S. Method development: J. D. S., T. W. S., and E. M. S. Sample collection/processing: J. D. S., S. B., A. P., C. R. C., D. W., C. H. B., and B. J. Data collection and analysis: J. D. S., V. K., B. K., S. B., A. P., C. R. C., C. A. R., and E. M. S. Writing–original draft: J. D. S. and E. M. S. Writing–review and editing: J. D. S., B. K., T. W. S., E. J. A., N. R., M. H. C., C. A. R., P. A., and E. M. S. Supervision and project administration: B. J. and E. M. S.

***Financial support***. This work was supported by the Infectious Diseases Clinical Research Consortium (IDCRC) through the National Institute of Allergy and Infectious Diseases (NIAID) of the NIH, under award number UM1AI148684 as an IDCRC Early Career Investigator Pilot Award to E. M. S. Mass spectrometry–based glycomics analysis was performed at the Complex Carbohydrate Research Center and was supported in part by the NIH-funded R24 grant (R24GM137782) to P. A. This work used samples and data from the Adaptive COVID-19 Treatment Trial (ACTT-1) trial (doi:10.1056/NEJMoa2007764), which was sponsored and primarily funded by NIAID. This trial has been funded in part with federal funds from NIAID and the National Cancer Institute, NIH, under contract HHSN261200800001E 75N910D00024, task order number 75N91019F00130/75N91020F00010, and by the Department of Defense, Defense Health Program. This trial has been supported in part by NIAID under award numbers UM1AI148684, UM1AI148576, UM1AI148573, UM1AI148575, UM1AI148452, UM1AI148685, UM1AI148450, and UM1AI148689. The trial has also been funded in part by the governments of Denmark, Japan, Mexico, and Singapore. The trial site in South Korea received funding from the Seoul National University Hospital. Support for the London International Coordinating Centre was also provided by the UK Medical Research Council (MRC_UU_12023/23). The sites and investigators involved with collection of the samples during the ACTT-1 trial are noted in the original manuscript.
